# Pregnancy and Neonatal Outcomes After Exposure to Alprazolam in Pregnancy

**DOI:** 10.3389/fphar.2022.854562

**Published:** 2022-04-25

**Authors:** Hyunji Lee, Jae-Whoan Koh, Young-Ah Kim, Kyoung-Chul Chun, Jung Yeol Han, Jong Hee Hwang, June-Seek Choi, Sung Hong Joo, Hye-Young Kwon

**Affiliations:** ^1^ Korean Mother-Safe Counselling Center, Pregnancy and Breastfeeding Medicines Information Center, Seoul, Korea; ^2^ Department of Obstetrics and Gynecology, Inje University Ilsan Paik Hospital, Goyang, Korea; ^3^ Department of Pediatrics, Inje University Ilsan Paik Hospital, Goyang, Korea; ^4^ Department of Obstetrics and Gynecology, CHA Gangnam Medical Center, CHA University, Seoul, Korea; ^5^ Department of Obstetrics and Gynecology, National Medical Center, Seoul, Korea; ^6^ Division of Biology and Public Health, Mokwon University, Daejeon, Korea

**Keywords:** anxiety disorders, low birth weight (LBW), spontaneous abortion (SA), alprazolam (Alp), pregnancy

## Abstract

Alprazolam is a commonly prescribed benzodiazepine for anxiety or panic disorder, even in pregnant women. Information on the safety of alprazolam during pregnancy is insufficient. We aimed to evaluate pregnancy and neonatal outcomes after exposure to alprazolam during pregnancy. A prospective study was conducted on 725 pregnancies from January 2000 to December 2019. Participants were recruited through the Korean Mother-Safe Program, a service providing information on drug-induced teratogenic risk during pregnancy and breastfeeding. Exposed (N = 96) and non-exposed (N = 629) women to alprazolam during pregnancy were selected and followed-up until delivery. Pregnancy outcomes, including spontaneous abortion, still birth, low birth weight (LBW), preterm birth, Apgar score (at 1 and 5 min), and malformations were measured and compared. Multivariable logistic regression was performed to examine the association between alprazolam exposure and outcomes. The mean age was 32.9 (SD 4.0) years in the alprazolam-exposed group and 31.8 (SD 3.8) years in the unexposed group (*p* = 0.008). The alprazolam exposure group demonstrated a significantly higher likelihood of pregnancy and neonatal outcomes: spontaneous abortion (OR = 2.38; 95% CI 1.20–4.69), LBW (OR = 3.65; 95% CI 1.22–11.00), and Apgar score at 1 min ≤ 7 (OR = 2.19; 95% CI 1.02–4.67). There was no significant difference in congenital abnormalities between the exposure and non-exposure groups. Our findings confirmed that alprazolam exposure during pregnancy was significantly associated with adverse pregnancy and neonatal outcomes, including spontaneous abortion, low birth weight, and Apgar score at 1 min ≤ 7. Alprazolam during pregnancy should be appropriately regulated and monitored.

## Introduction

Alprazolam is one of the most commonly prescribed benzodiazepines for anxiety or panic disorder and is increasingly prescribed in pregnant women (Ait-Daoud et al., 2018; Tinker et al., 2019). It has been confirmed in animal studies that alprazolam may cause teratogenic effects. Oral cleft lip and palate as well as limb anomalies were observed in rat offspring (Takzare et al., 2011). Moreover, studies using rodents have shown behavioral abnormalities associated with prenatal exposure to alprazolam (Jaiswal, 2002; Rayburn et al., 2002). On the other hand, human studies have revealed that the use of benzodiazepines during pregnancy is not expected to increase the risk of congenital malformations (Czeizel et al., 1992; Eros et al., 2002). Nevertheless, a meta-analysis of 14 studies on pregnancy and delivery outcomes after exposure to benzodiazepines, concluded that exposure to benzodiazepines was associated with an increased risk of spontaneous abortion (OR = 1.86; 95% CI 1.43–2.42), preterm birth (OR = 1.96; 95% CI 1.25–3.08), low birth weight (OR = 2.24; 95% CI, 1.41–3.88), low Apgar score (OR = 2.19; 95% CI 1.94–2.47), and neonatal intensive care unit (NICU) admission (OR = 2.61; 95% CI 1.64–4.14), and induced abortion among pregnant women (Grigoriadis et al., 2020). Furthermore, Källén et al. (2013) reported that exposure during pregnancy to alprazolam, a derivative of benzodiazepines, was associated with a 1.97-fold increased risk of congenital malformations. Despite these risks, some pregnant women still need to take alprazolam to avoid the harmful effects of mental illness on maintaining pregnancy (Bais et al., 2020). According to American College of Obstetricians and Gynecologists (ACOG) guidelines (Armstrong, 2008), it is recommended to prescribe alprazolam (benzodiazepines) to those with psychiatric illness during pregnancy after considering its risks versus benefits. Although the use of psychotropic medications in these women is a concern because of the risks of adverse perinatal and postnatal outcomes, advising these women to discontinue medication presents new risks associated with untreated or inadequately treated mental illness, such as poor adherence to prenatal care, inadequate nutrition, and increased alcohol and tobacco use. Ideally, decisions about psychiatric medication use during and after pregnancy should be made before conception. Additionally, the use of a single medication is preferred over multiple medications.

Since 2008, the Korean Ministry of Drug and Food Safety (MDFS) has published a list of drugs contraindicated for use in pregnant women because of significant harm to the fetus. The most recently published list published by the MDFS, dated August 31, 2021, contains 1,078 drugs that are contraindicated during pregnancy. Of these, 131 were classified as Class 1, which should not be used during pregnancy. The rest fell into Class 2, and their use is prohibited in principle as the risks outweigh the benefits, but exceptions can be made at the prescriber’s decision. Benzodiazepines, including alprazolam and triazolam, have been reported as Class 2 (Ministry of Drug and Food Safety, 2021). However, the most commonly prescribed drugs for sedative-hypnotics in South Korea were benzodiazepines such as alprazolam and diazepam (Lee et al., 2019). Alprazolam is one of the most commonly prescribed medications for anxiety (Lee et al., 2015; KS, 2019), including for pregnant women.

Nevertheless, there is a dearth of studies pertaining to the impact of alprazolam itself on pregnancy and neonatal outcomes. Therefore, this study aimed to investigate the use of alprazolam, a class of benzodiazepines, among pregnant women in Korea and its impact on pregnancy and neonatal outcomes in a prospective cohort setting.

## Materials and Methods

Since 2000, a pregnancy exposure registry has been established from the Korean Mother-Safe Program, funded by the Ministry of Health and Welfare. The registry has enrolled pregnant women participating in the Mother-Safe Program, and their health information and information on the newborn baby have been collected. The Mother-Safe Program is a teratogen information service that provides evidence-based information on the safety and risk of exposure to prescription medicines, smoking, chemicals, and radiation during pregnancy and breastfeeding, which is operated by the Cheil General Hospital and Women’s Healthcare Center in Seoul.

**FIGURE 1:**
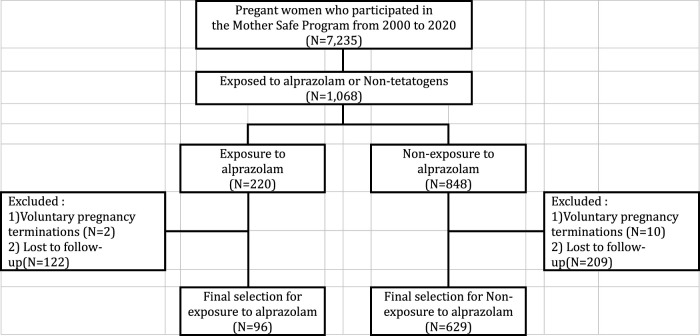
Selection of study participants.

Study subjects were selected among pregnant women who participated in the Korean Mother-Safe Program. A total of 7,235 women seeking services for their pregnancies from January 2000 to December 2019 were registered. Of these, 1,068 women were selected as they were exposed to alprazolam or non-teratogenic drugs including acetaminophen and chlorpheniramine during pregnancy. We excluded cases of voluntary pregnancy terminations (N = 12) and those who were lost to follow-up (N = 331). Finally, a total of 725 pregnant women were selected and classified into two groups: alprazolam exposure and non-exposure groups. They were followed up until their delivery to check their pregnancy and neonatal outcomes, including spontaneous abortion, stillbirth, low birth weight (LBW, < 2,500 g), preterm birth (< 37 weeks), Apgar score at 1 and 5 min (7 or less), and malformations (Figure 1).

Socio-demographic information [i.e., age, body mass index (WHO Expert Consultation, 2004), education, and occupation] of the study population was recorded. Information on exposure to other teratogens, such as alcohol intake, smoking, and radiation, was obtained from each participant. In addition, the daily dose, duration of alprazolam administration, and the reason for taking it were asked in the exposed group. Additional medications taken during pregnancy were also asked at the time of counselling. These variables were considered as covariates to estimate the impact of alprazolam exposure on pregnancy and neonatal outcomes.

Categorical variables were expressed as frequencies and percentages, and continuous variables as means and standard deviations (SDs). Chi-square test for categorical variables and Student’s *t*-test for means were performed. The Mann-Whitney median test was used for skewed distribution. Fisher’s exact test for categorical variables was used when appropriate. To examine the association between alprazolam exposure during pregnancy and pregnancy and neonatal outcomes, a multivariable logistic regression was performed using SPSS version 26.0 (IBM, SPSS Statistics, Armonk, NY, United States).

### Ethical Approval

The study was approved by the Institutional Review Board at the Cheil General Hospital and Women’s Healthcare Center, Seoul, Korea (IRB No: CGH-IRB-2010–21). Written informed consent was obtained from all participants. All participants were fully anonymized.

## Results

### General Characteristics

As tabulated in Table 1, the exposed group was relatively older (32.9 SD 4.0 vs. 31.8 SD 3.8, *p* = 0.008) than the unexposed group. Those aged 35 years or older accounted for 29.2% of the exposed group and 19.6% of the unexposed group (*p* = 0.031). Alcohol consumption was 27.1% (26 out of 96) of women exposed to alprazolam and 39.7% (250 out of 629) in the unexposed group (*p* < 0.001). The gestational age at the time of enrollment of the exposed and unexposed groups was 8.8 weeks (SD 4.6) and 9.0 weeks (SD 3.8), respectively, which was not significantly different (*p* = 0.696). Other characteristics were not significantly different between the two groups.

**TABLE 1:** Basic characteristics of participants.

Variables				Alprazolam Exposure Group (N = 96)		Alprazolam non-exposure Group (N = 629)		*P*-value
				N	%	N	%	
Age (years), Mean (SD)				32.9	(4.0)	31.8	(3.8)	0.008
Age			<35	68	70.8	506	80.4	0.031
			≥ 35	28	29.2	123	19.6	
Gravidity, mean (SD)				2.2	(1.3)	2.2	(1.2)	0.928
Parity			<1	48	50.0	276	43.9	0.261
			≥1	48	50.0	353	56.1	
BMI^a^			<18.5 (kg/m^2^)	19	19.8	112	17.9	0.666
			≥18.5 and <23 (kg/m^2^)	60	62.5	378	60.5	
			≥23 (kg/m^2^)	17	17.7	135	21.6	
Alcohol intake				26	27.1	250	39.7	0.017
Smoking				11	11.5	56	8.9	0.421
Radiation				17	17.7	91	14.5	0.406
Education Level			High school or less	7	7.3	45	7.2	0.568
			College	2	2.1	16	2.5	
			University	41	42.7	223	35.5	
			No answer	46	47.9	345	54.8	
Occupation			White collar	39	40.6	282	44.8	0.203
			Blue collar	4	4.2	51	8.1	
			Housewife	53	55.2	296	47.1	
Gestational weeks on counselling time (Mean, SD)				8.8 ± 4.6		9.0 ± 3.8		0.696
Number of co-exposure drugs (Mean, SD)				9.8 ± 8.5		9.7 ± 8.1		0.923

a BMI, Body Mass Index. The pre-pregnancy BMI was classified according to Asia-specific standards (WHO Expert Consultation, 2004).

### Use of Alprazolam During Pregnancy

Among the exposure group, the main reasons for taking alprazolam were irritable bowel syndrome (IBS) (20.8%), followed by depression (16.7%), and respiratory diseases, including common cold (12.5%) (Table 2). Given that the defined daily dose (DDD) of alprazolam (ATC code: N05BA12) by World Health Organization (WHO) is 1 mg [(WHO), 2021], the actual daily doses of alprazolam of the exposure group ranged from 0.13 to 0.50 mg (median 0.25 mg), which is lower than the assumed average maintenance dose per day for a drug used for its main indication in adults. Cumulative doses during pregnancy ranged from 1.00 mg/kg to 61.00 mg/kg (median 16.00 mg/kg). The mean gestational age (GA) when they last took alprazolam was GA 4 weeks 1 day (from 1 week 2 days–12 weeks 3 days). Pregnant women in the exposure group reported that they had taken eight drugs (0–37 drugs) on average, together with alprazolam (Table 2).

**TABLE 2:** Description of alprazolam use in pregnancy (N = 96).

Indications	N	(%)
Irritable bowel syndrome	20	20.8
Depression	16	16.7
Respiratory diseases including common cold	12	12.5
Panic disorder	11	11.5
Other neuropathy including migraine	11	11.5
Obesity	9	9.4
Anxiety	7	7.3
Insomnia	7	7.3
Overactive bladder	3	3.1
Alprazolam use	Median	Min- Max
Gestational age at last dose^a^	4 W 1 D	1 W 2 D∼ 12 W 3 D
Daily doses (mg)	0.25	0.13–0.50
Cumulative dose during pregnancy (mg/kg)	16.00	1.00–61.00

a Additional doses were not followed up for the remainder of the pregnancy after teratogenic risk counselling.

W, Week(s); D, Day(s).

### Pregnancy and Neonatal Outcomes

As shown in Table 3, pregnant women gave birth at GA 38.8 weeks (SD 2.1) in the exposure group and GA 39.3 (SD 1.8) in the non-exposure group (*p* = 0.012). Significant differences between the two groups were found in all measures except stillbirth, abnormalities, and birth weight. The difference in the Apgar score at 5 min between the two groups was marginally significant (*p* = 0.074). Overall, adverse pregnancy and neonatal outcomes were more frequent in the exposure group than in the non-exposure group: spontaneous abortion (14.6 vs. 6.0%, *p* = 0.003), low birth weight (LBW) (7.5 vs. 2.1%, *p* = 0.008), preterm birth (8.5 vs. 3.8%, *p* = 0.048), Apgar score at 1 min (18.0 vs. 9.6%, *p* = 0.006). were significantly more prevalent in the exposure group than in the non-exposure group.

**TABLE 3:** Pregnancy and neonatal outcomes.

Outcomes	Exposure (N = 96)		Non-exposure (N= 629)		*P*-value	Crude ORs (95% CI)	Adjusted ORs^a^ (95% CI)
Gestational age at birth (weeks), Mean (SD)	38.8	(2.1)	39.3	(1.8)	0.012	—	—
Spontaneous Abortion, N (%)	14	(14.6)	38	(6.0)	0.003	2.66 (1.38, 5.11)	2.38 (1.20, 4.69)
Stillbirth, N (%)	1	(1.0)	2	(0.3)	0.347		
Low birth weight (<2,500 g), N (%)	6	(7.5)	10	(2.1)	0.008	3.74 (1.32, 10.59)	3.65 (1.22,11.00)
Birth weight (g), Mean (SD)	3,239	(580)	3,312	(450)	0.278	—	—
Preterm birth (<37 weeks), N (%)	7	(8.5)	22	(3.8)	0.048	2.38 (0.98,5.76)	2.27 (0.92,5.60)
Apgar score at 1 min, N (%)	11	(18.0)	45	(9.6)	0.044	2.07 (1.01,4.26)	2.19 (1.02,4.67)
Apgar score at 5 min, N (%)	3	(4.9)	6	(1.3)	0.074	3.98 (0.97,16.36)	3.68 (0.85,16.00)
Abnormality, N (%)	3^b^	(3.1)	17^c^	(2.7)	0.724	—	—

a Adjusted for age, parity, body mass index, alcohol intake, smoking, education, and occupation.

b Polydactyly of the left foot (1), ankyloglossia (1), cholesteatoma on the roof of the mouth (1).

c Micropenis (1), Atrial septal defect with patent ductus arteriosus (1), Cyst of fetal right kidney (1), Right hydronephrosis with right scrotal hydrocele (1), Necrotizing enterocolitis with perforation (2), Congenital cystic adenomatoid malformation of right lung (1), Discrepancy in testis size (1), Both club foot (1), Right club foot with persistent right umbilical vein (1), Bowel obstruction (1), Atonic seizure (2), Wrist drop of left hand (1), Nasolacrimal duct obstruction with ptosis of both eyes (1), Large hemangioma of face (1), Left hydrocephaly (1).

Although the difference in the frequency of congenital abnormalities was not significant (3.1 vs. 2.7%, *p* = 0.739), three cases of abnormalities including polydactyly of the left foot, ankyloglossia, and cholesteatoma on the roof of the mouth were detected in the fetus of the alprazolam-exposed group.

The association between alprazolam exposure and pregnancy and neonatal outcomes was examined after controlling for confounding variables. As a result, those exposed to alprazolam during pregnancy were 2.38 (95% CI 1.20–4.69) times more likely to experience spontaneous abortion, 3.65 (95% CI 1.22–11.00) times more likely to have a baby with LBW, and 2.19 (95% CI 1.02–4.67) times more likely to have Apgar score at 1 min < 7 than those who were not exposed (Table 3).

## Discussion

This study investigated the association between alprazolam exposure during pregnancy and adverse pregnancy and neonatal outcomes. The results confirmed that exposure to alprazolam during pregnancy was significantly associated with an increased risk of spontaneous abortion, low birth weight, and Apgar score at 1 min ≤ 7. These findings were consistent with those of previous studies on benzodiazepines (Grigoriadis et al., 2020).

Regarding preterm birth, Grigoriadis et al. (2020)’s meta-analysis reported that exposure to benzodiazepine during pregnancy was 1.96 (95% CI 1.25–3.08) times more likely to lead to preterm birth than non-exposure. The risk of preterm birth in our study, yet statistically insignificant, was increased 2.27-fold compared to non-exposure to alprazolam (OR 2.27 95% CI 0.92–5.60). Further studies with a larger scale are needed to obtain robust results.

The causes of spontaneous abortion are multiple etiologies including chromosomal abnormality, maternal thrombophilic disorders, immune dysfunction and various endocrine disturbances as well as life style factors such as alcohol, smoking and obesity (Larsen et al., 2013).

Although the pathophysiology of benzodiazepines causing spontaneous abortion is unknown, several studies have confirmed that exposure to benzodiazepines is significantly associated with spontaneous abortion (Ornoy et al., 1998; Ban et al., 2012; Sheehy et al., 2019). This can be explained by the following characteristics: benzodiazepines freely cross the placenta and may accumulate in the embryo and fetal tissues, causing adverse effects. Benzodiazepines not only act as agonists of GABA receptors in the central nervous system (CNS), but also bind to peripheral tissues and may cause steroidogenesis and cell proliferation (Sheehy et al., 2019).

The results of previous studies on the association between alprazolam exposure during pregnancy and congenital malformations are controversial. A study (Tinker et al., 2019) concluded that alprazolam was associated with an increased risk of anophthalmia or microphthalmia, esophageal atresia or stenosis, hypospadias, and atrioventricular septal defect (AVSD). In contrast, Bellantuono and colleagues (Bellantuono et al., 2013) found an elevated risk of spontaneous abortion, but no higher risk of any malformation in live-born children. A meta-analysis on the fetal safety of benzodiazepines concluded that exposure to benzodiazepines was only associated with an increased risk of oral cleft (Enato et al., 2011). In our study, no significant differences in congenital malformations were found between the two groups. The three cases with malformations in the exposed group were polydactyly of the left foot, ankyloglossia, and cholesteatoma on the roof of the mouth. However, this study with a relatively small sample size (N = 96 in exposure group) may not have the statistical power to expose such a small effect, possibly resulting in a type II error. Thus, our findings cannot be confirmatory. On the other hand, there was no consistency in the types of malformations in the non-exposures.

Our study found that the most common indication for alprazolam administration in pregnancy was irritable bowel syndrome (IBS) (20.8%). Maternal IBS is associated with an increased risk of miscarriage and ectopic pregnancy, but not with preeclampsia or stillbirth (Moosavi et al., 2021). Accordingly, maternal IBS in early pregnancy needs to be treated without compromising pregnancy outcomes.

Additionally, insomnia during pregnancy is related to adverse pregnancy outcomes such as prenatal depression, gestational diabetes, pre-eclampsia, abnormal length of labor, cesarean delivery, alteration in fetal growth, and preterm birth (Palagini et al., 2014). Although sleep disorders need to be managed, alprazolam in pregnancy might be related to spontaneous abortion, low birth weight, preterm birth, and low Apgar score. Therefore, non-pharmacological management such as maintaining good sleep hygiene should be considered first, and if necessary, prescribing drugs, including zolpidem, is recommended (Creeley & Denton, 2019).

The management of pregnant women with chronic diseases including IBS, depression, panic disorder, anxiety, insomnia, and others indicated that the use of alprazolam in pregnancy should be multidisciplinary, and dietary modifications and pharmacologic options such as alprazolam should be considered at the same time.

The advantages of this study are as follows. First, as it was prospectively conducted, the association between exposure and pregnancy outcomes was more clearly obtained without recall bias. Next, although there were various studies such as systematic reviews or meta-analyses of benzodiazepine and pregnancy outcomes, the studies were not conducted on each single drug of the benzodiazepine class. In addition, as a single agent, alprazolam was investigated in a large study (N = 411), published in 1992 (St Clair & Schirmer, 1992), where there were no data on birth weight, preterm birth, and Apgar score. However, our study was conducted with a larger sample size (N = 725) with data on low birth weight, preterm labor, and Apgar score.

Our study also has some limitations. First, our results may have been overestimated due to an indication bias. Alprazolam was approved in South Korea for the treatment of generalized anxiety disorders, panic disorders, and anxiety/depression/sleep disorders in psychosomatic disorders such as irritable bowel syndrome, gastroduoduenal ulcer, and ataxia. Naturally, these conditions are more likely to be prevalent in the exposure group. However, the small sample size for alprazolam exposure group (N = 96) in this study made it difficult to enroll pregnant women who did not take alprazolam but had the relevant indications. Given that a meta-analysis showed a significant association between maternal depression and preterm birth (OR = 1.37; 95% CI 1.04–1.81) but no significant association for LBW, GA, and Apgar score at 1 and at 5 min (Grigoriadis et al., 2013), the confounding effect of maternal depression on pregnancy and neonatal outcomes may be negligible. Conversely, Rejno et al. (2019) revealed that maternal depression has been associated with shorter gestation (−0.29 weeks, *p* < 0.001) and lower birth weight (−54 g, *p* < 0.001). Therefore, further studies fully controlling confounders should be made. Second, as the study participants were recruited from those who visited Cheil General Hospital and Women’s Healthcare Center, a tertiary hospital for high-risk pregnant women located in Seoul, a sampling bias in the recruitment of study participants was embedded in our study. Thus, our results may not be generalizable to the entire population. Third, as shown in Figure 1, when selecting the study participants, the exposure group (55.5%) had higher rate of loss to follow-up than the non-exposure group (24.7%), which may result in biased estimates. This is a major limitation of our prospective cohort study, and cautious interpretation of the results is required. Fourth, although it is possible that co-exposure to other drugs taken simultaneously affected adverse pregnancy outcomes, we could not control this because no further analysis on co-exposure drugs could be made. However, the co-exposure drugs did not include any pregnancy-contraindicated drugs or drugs harmful to the fetus. Lastly, this study has uncertainty regarding the duration of alprazolam use during pregnancy after teratogen counseling and the lack of evaluation of newborns’ long-term outcomes, including neurodevelopment.

Despite these limitations, to the best of our knowledge, our study is the first attempt to investigate the impact of alprazolam exposure on pregnancy and neonatal outcomes in Korea. Consistent with some known findings, our study revealed that alprazolam exposure during pregnancy was significantly associated with adverse pregnancy and neonatal outcomes such as spontaneous abortion, low birth weight, and Apgar score at 1 min ≤ 7.

Further studies are needed that consider predisposing diseases requiring alprazolam prescription during pregnancy or long-term observation of neurodevelopment in neonates.

## Data Availability

The original contributions presented in the study are included in the article/Supplementary Material, further inquiries can be directed to the corresponding authors.
