# Corrigendum: Pregnancy and Neonatal Outcomes After Exposure to Alprazolam in Pregnancy

**DOI:** 10.3389/fphar.2022.934265

**Published:** 2022-08-05

**Authors:** Hyunji Lee, Jae-Whoan Koh, Young-Ah Kim, Kyoung-Chul Chun, Jung-Yeol Han, Jong Hee Hwang, June-Seek Choi, Sung-Hong Joo, Hye-Young Kwon

**Affiliations:** ^1^ Korean Mother-Safe Counselling Center, Pregnancy and Breastfeeding Medicines Information Center, Seoul, South Korea; ^2^ Department of Obstetrics and Gynecology, Ilsan Paik Hospital, Inje University College of Medicine, Busan, South Korea; ^3^ Department of Pediatrics, Ilsan Paik Hospital, Inje University College of Medicine, Busan, South Korea; ^4^ Department of Obstetrics and Gynecology, CHA Gangnam Medical Center, CHA University, Seoul, South Korea; ^5^ Department of Obstetrics and Gynecology, National Medical Center, Seoul, South Korea; ^6^ Division of Biology and Public Health, Mokwon University, Daejeon, South Korea

**Keywords:** anxiety disorders, low birth weight (LBW), spontaneous abortion (SA), alprazolam (Alp), pregnancy

Hye-Young Kwon was not included a corresponding author in the published article by mistake. As stated, both Jung-Yeol Han and Hye-Young Kwon both equally contributed to the article as corresponding authors.

The authors apologize for this error and state that this does not change the scientific conclusions of the article in any way. The original article has been updated.

